# Cardioimmunologic response patterns after an acute heart failure event: Design and first results of AHF‐ImmunoCS

**DOI:** 10.1002/ehf2.70005

**Published:** 2025-10-30

**Authors:** Niklas Beyersdorf, Boshra Afshar, Dora Pelin, Maximilian Bauser, Elisa Kaiser, Janna Lamers, Jannika Pätkau, Mairin Heil, Dennis Göpfert, Hanna Hepp, Wafaa Al Hassan, Thomas Kerkau, Fabian Kerwagen, Roland Jahns, Valerie Boivin‐Jahns, Stefan Frantz, Gustavo Ramos, Ulrich Hofmann, Stefan Störk, Caroline Morbach

**Affiliations:** ^1^ Institute for Virology and Immunobiology University of Würzburg Würzburg Germany; ^2^ Department Clinical Research and Epidemiology, Comprehensive Heart Failure Center University Hospital Würzburg Würzburg Germany; ^3^ Department of Medicine I University Hospital Würzburg Würzburg Germany; ^4^ Interdisciplinary Bank of Biological Materials and Data Würzburg (IBDW) University Hospital Würzburg Würzburg Germany; ^5^ Institute for Pharmacology and Toxicology University of Würzburg Würzburg Germany

**Keywords:** adaptive immune response, B cell, heart‐reactive antibody, prognosis, worsening heart failure

## Abstract

**Aims:**

We have previously shown that patients who develop heart‐reactive antibodies (HRAs) de novo after a heart failure (HF) hospitalization are at increased risk of adverse outcomes, lending weight to the hypothesis that B cells may play a pivotal role in HF progression. We therefore aim to further elucidate the adaptive immune response to an acute HF event, with a particular focus on the factors that lead to incident HRAs and their relation to worsening cardiac function and prognosis.

**Methods and results:**

The Acute Heart Failure Immunomonitoring Cohort Study (AHF‐ImmunoCS) is a prospective monocentric cohort study. Patients are enrolled consecutively during hospitalization for AHF and undergo detailed phenotyping at baseline and at 6‐week, 6‐, 12‐ and 18‐month follow‐up visits. Patient sera are screened for HRAs by immunofluorescence testing (IFT) as well as by using defined cardiac antigens immobilized on beads. By 31 December 2023, we had included 259: 38% women, mean age 72 (SD 13) years, 37% de novo HF, median left ventricular ejection fraction 50 (quartiles 35, 56) %. Preliminary data of the first 59 patients (42% women, mean 72 (14) years) showed that 80% of patients exhibiting seroconversion had done so within 6 weeks.

**Conclusion:**

AHF‐ImmunoCS is enrolling a representative cohort of HF patients. Our preliminary data confirm that seroconversion to HRAs occurs early after hospitalization for HF in a subgroup of patients. The full study can be expected to clarify how changes in HRA profiles relate to prognosis and may pave the way for novel immunotherapeutic approaches to acute heart failure.

## Graphical abstract

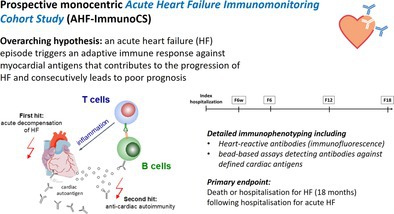

## Introduction

### Background

Acute heart failure (AHF) is associated with high morbidity and mortality and, when compared with chronic stable heart failure (HF), the mortality risk remains 5–10 times higher for months after an episode of hospitalization.^1^, ^2^, ^3^ Despite emerging therapeutic options, cardiac function tends to progressively deteriorate over time, with frequently observed worsenings after episodes of decompensation.^4^ Yet the underlying causes of AHF episodes, that is, the circumstances and factors triggering acute decompensation, as well as the determinants of HF progression, are still poorly understood.

It is a well‐established concept that AHF is accompanied by and/or induces a systemic inflammatory response^1^ that likely primarily reflects the activation of the innate immunity. Systemic concentrations of inflammatory biomarkers increase as HF worsens, and levels of C‐reactive protein and cytokines, like interleukin‐1β, predict death and correlate with exercise capacity in patients with HF.^1^ In addition, interleukin‐1β neutralization in clinically stable patients with a history of myocardial infarction reduced the risk of HF‐related hospitalization in the randomized CANTOS trial^5^ thus successfully transferring this concept from experimental studies to humans. These clinical data further demonstrated that innate inflammation promotes HF progression and emergency hospitalization due to AHF.

Only little is known about the role of B cells and adaptive immunity in HF. However, there is growing evidence that B cells might play a pivotal role in the development and progression of HF^6^ and, thus, might be potential targets for novel immunomodulatory therapies. Here, the RITA‐MI trial^7^ has shown that B cell depletion by a single dose of rituximab was safe in patients after myocardial infarction paving the way for further studies on efficacy.

B cells have several important physiological functions and are involved in virtually any form of immune response: on the one hand, in synergy with T cells, they present antigens and produce antibodies; on the other hand, they produce cytokines to modulate the function of other leukocytes.^6^ In the context of heart disease and HF, antibodies have been detected targeting a variety of heart‐associated antigens: receptors like the β1‐adrenergic (AR)^8^, ^9^ and the muscarinic type 2 receptor as well as structural and non‐structural myocardial proteins like troponin and myosin, but also ion channels like the Na‐K‐ATPase.^6^

In patients with HF with reduced ejection fraction (HFrEF), heart‐reactive autoantibodies (HRAs) were frequent^10^ and patients with end‐stage HF were found to exhibit myocardial deposition of IgG.^11^ Yet the significance of these findings is not fully established. Autoantibodies are considered to potentially have cardiotoxic effects^12^ and mediate myocarditis.^6^, ^13^ Consistently, small studies investigating immunoadsorption of serum antibodies in patients with dilated cardiomyopathy showed an improvement in cardiac function.^14^, ^15^

Animal experiments elucidated mechanisms of how autoantibodies can promote myocardial disease. Regarding HRAs in HFrEF, mechanistic insights were gained in rabbits. Experimentally inducing antibodies targeting cardiac proteins triggered morphological changes of the heart in rabbits concordant with cardiomyopathy and HFrEF.^16^ Further, several studies in animal models suggested also an antibody‐independent role of B cells in the establishment and progression of HF, for example, by monocyte recruitment and orchestration of the amplification of several immune cell types in the pericardial tissue.^6^ In contrast, analyses of a murine model of ischaemic HFrEF imply a potential protective effect against adverse remodelling of interleukin‐10 producing B cells in the pericardial tissue.^17^ In patients with HFrEF, higher frequencies of replicating^18^ and of regulatory B cells^19^ have been described. In line with this, a reduction in circulating B cells in some HFrEF patients seemed to improve their response to HF pharmacotherapy as shown in a small study.^20^ These data suggest a central, but still poorly defined, role of adaptive immunity in HFrEF.

Regarding HF with preserved ejection fraction (HFpEF), the available data suggest that B cells might be a key player in the development of this syndrome.^6^ In rabbits, immunization‐triggered HRA production against the β1‐adrenergic receptor (AR) induced left ventricular (LV) hypertrophy, a decrease in LV size, and an increase in LV pressure, suggesting that antibodies can modulate myocardial remodelling and potentially contribute to the development of HFpEF.^21^ Respective antibodies are able to stabilize β1‐AR in their activated form^22^; hence, the respective effect might be mediated by an HRA‐mediated alteration of β1‐AR signalling.^6^ Moreover, HRAs against the second extracellular loop of the β1‐adrenergic receptor serve as a model for specific immunotherapy in cardiac disease.^23^, ^24^

On the other hand, B cell depletion resulted in reduced LV hypertrophy and reduced collagen deposition in mice, thus ameliorating HF development,^25^ potentially mediated by decreased deposition of IgG_3_ in the myocardium and reduced collagen synthesis. Further, regulatory B cells were shown to reduce maladaptive changes in a murine model of pressure overload‐induced myocardial hypertrophy and fibrosis.^26^ In humans, proteomic analyses of serum from HFpEF patients showed up‐regulated markers compatible with activation of B cells.^27^ Further, data from the phase II PIROUETTE trial showed that the immunomodulatory small molecule pirfenidone reduced cardiac fibrosis.^28^

### Rationale

Data from animal models suggest a central role for B cells in the development and progression of HF.^6^ B cells can promote or inhibit cardiac fibrosis, hypertrophy, and contractile dysfunction, all of which contribute to the development and progression of HF.^6^ Molecularly, the key function of B cells is to mediate antibody secretion. Antibody isotype class switching as well as affinity maturation due to somatic hypermutation heavily depends on the interaction with CD4^+^ T helper cells. Therefore, studying HRA responses also provides for an indirect read‐out of T cell immunity. Independent of the exact pathophysiological role of B cells in HF and HRAs can serve as biomarkers for heart‐specific B cell activation and autoimmunity.

**Figure 1:**
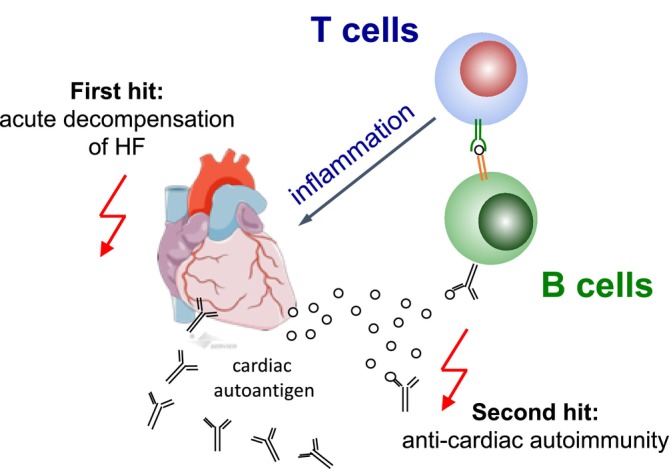
We hypothesize that the innate immune response immediately triggered by acute decompensation of HF (‘first hit’) provides the pro‐inflammatory context, by activating antigen‐presenting cells, required to break tolerance and induce adaptive immunity to cardiac antigens (‘second hit’) thus contributing to disease progression and adverse prognosis.

Although first analyses in patients with HF point towards an adverse role of HRAs, we do not yet know how adaptive immune responses to cardiac antigens contribute to the clinical progression of HF following AHF in humans. Mechanistically, the innate immune response immediately triggered by acute decompensation of HF (‘first hit’, *Figure* *1*) provides the pro‐inflammatory context by activating antigen‐presenting cells required to break tolerance and induce adaptive immunity to cardiac antigens (‘second hit’). While innate immune responses are short‐lived, adaptive immune responses are lasting and create immunological memory, and may as such particularly contribute to the worsening of heart function and prognosis in the mid‐ and long‐term following AHF.

In a first approach to test our hypothesis, we aimed to assess the prevalence and titre course of HRAs in AHF patients and to determine their prognostic potential. From a local prospective cohort study (Acute Heart Failure Registry) following *n* = 1000 consecutive patients hospitalized for AHF, we retrospectively selected a carefully stratified sample consisting of 48 patients.^29^, ^30^ Strata were male vs female, chronic vs de novo manifestation of HF, and reduced left ventricular ejection fraction (LVEF <40%, HFrEF) vs preserved LVEF (LVEF ≥50%, HFpEF). HRAs were determined in prospectively collected blood samples taken at baseline and six‐month follow‐up (F6). There was a high prevalence of HRAs in patients hospitalized for AHF (*n* = 21; 45%), which appeared to be independent of sex, HF phenotype, and mode of presentation (all *P* > 0.05). We found an increase in the prevalence of HRAs from baseline to F6 (*n* = 36, 77%; *P* < 0.001) and a higher prevalence of HRAs in HFpEF (*n* = 21; 88%) than in HFrEF at F6 (*n* = 14, 61%; *P* = 0.036). Prognosis differed according to HRA status and patients with newly developed HRAs at F6 had a significantly worse prognosis in the subsequent 12 months compared to the other patients.^29^ Our preliminary results, thus, strengthen the hypothesis that AHF may induce an adverse adaptive immune response and support further research regarding the role of the adaptive immune system in the progression of HF. To the best of our knowledge, AHF‐ImmunoCS is the first prospective study to investigate the *de novo* induction of HRAs after acute decompensation of HF and their prognostic value. We therefore believe that AHF‐ImmunoCS has the potential to significantly advance our understanding of the contribution of HRAs to HF progression.

### Hypothesis and main research questions

We follow the overarching hypothesis that an AHF episode triggers an adaptive immune response against myocardial antigens that contributes to the progression of HF and consecutively leads to poor prognosis (*Figure* *1*). We will address the following research questions:

1. What are the prevalence and determinants of HRAs (binding to heart tissue sections, specific for defined antigens) in AHF patients at baseline and over time?
2. Can we detect cardiac antigen‐specific B cells and T cells in AHF patients?
3. What are the characteristics and determinants (cytokine secretion, cellular immunophenotypes) of the adaptive immune response to an AHF episode?
4. Is there evidence that newly generated HRAs following an AHF episode are associated with the progression of cardiac impairment and the prognosis in these patients?

## Study design and first results

### Overall design

The Acute Heart Failure Immunomonitoring Cohort Study (AHF‐ImmunoCS) is a prospective monocentric cohort study embedded in a local Collaborative Research Centre CRC 1525 uniting clinical and basic researchers. The study adheres to the principles of Good Clinical Practice and is covered by an ethical vote of the local Ethics Commission (#112/21). All patients sign a written informed consent prior to study start. Patients are consecutively enrolled at the time of hospitalization for acute heart failure (AHF) and phenotyped in detail during the index hospitalization. The observation period after discharge from index hospitalization covers an 18‐month period (*Table* *1*).

**Table 1:** Schedule of assessments and procedures for each patient participating in the AHF‐ImmunoCS

Month	Index AHF admission	Follow‐up				
	0	F6w	F6	F12	F18
Visit	1	2	3	4	5
Patient informed consent	X					
Patient ID, Log‐in procedure in Medical Information System	X					
Demographics	X					
Type and mode of admission	X					
Pharmacotherapy (prior to admission)	X					
Rescue measures (including current pharmacotherapy)	X					
Clinical history review (cardiovascular and general)	X					
Time spent in hospital and respective wards	X					
Discharge information and diagnosis	X					
Clinical presentation (NYHA, signs and symptoms of HF)	X	X	X	X	X
Biometry (height, weight and blood pressure)	X	X	X	X	X
Detailed transthoracic echocardiogram	X	X	X	X	X
Electrocardiogram	X	X	X	X	X
6‐minute walk test	‐	X	X	X	X
Routine laboratory measurements^a^	X	X	X	X	X
Biomaterial collection^b^	X	X	X	X	X
Complications during index hospitalization and new co‐morbidities	X	X	X	X	X
Pharmacotherapy	X	X	X	X	X
Additional treatment options	X	X	X	X	X
Re‐hospitalization discharge information^c^		X	X	X	X
Survival status and clinical outcomes		X	X	X	X

AHF, acute heart failure; F12, 12‐month follow‐up; F18, 18‐month follow‐up; F6, six‐month follow‐up; F6w, six‐week follow‐up; HF, heart failure; ID, identifier; NYHA, New York Heart Association.

^a^ Sodium, potassium, calcium, chloride, creatinine, phosphate, urea, uric acid, iron, ferritin, transferrin saturation, total protein, albumin, total cholesterol, low densitiy cholesterol, high density cholesterol, triglycerides, glucose, thrombocytes, leucocytes, erythrocytes, haemoglobin, haematocrit, N‐terminal pro B‐type natriuretic peptide, alanin aminotransferase, aspartate aminotransferase, gamma glutamyltransferase, creatinine kinase (CK), CK‐MB and high‐sensitive troponin.

^b^ Study biomaterials (serum, plasma and cells) are sampled and stored once during enrolment (prior to discharge), and at every follow‐up visit. From these biomaterials, we will determine heart reactive antibodies.

^c^ Heart failure‐related hospitalization following index admission discharge.

The study has been registered (ISRCTN18181364).

### Patient population

Consecutive patients hospitalized for AHF will be included if they fulfill all the following criteria:

- Hospitalization for AHF (as considered by the treating physician)
- Age ≥ 18 years
- Written informed consent
- Willingness to attend planned follow‐up visits
- Life‐expectancy ≥ 6 months

Patients fulfilling **any** of the following criteria are not eligible for inclusion in this study:

- High output HF (e.g., systemic arteriovenous fistula, hyperthyroidism and sepsis)
- Cardiogenic shock (requiring inotropes or circulatory support)
- High urgency listing for a heart transplant

### Patient phenotyping

During index hospitalization, patients are phenotyped in detail, including:

- medical history prior to enrolment including onset of HF, cardiac and non‐cardiac disorders and comorbidities, pharmacotherapy;
- medical information during index hospitalization including diagnostic and therapeutic procedures, pharmacotherapy, psychometric assessment, complications, outcomes;
- prior to discharge, a basic clinical data set is collected including height, weight and blood pressure, and venous blood is drawn for both immediate analysis and long‐term storage.

## Follow‐up period

Six weeks as well as 6 months, 12 months, and 18 months after enrolment, patients are re‐invited to an outpatient visit at the Comprehensive Heart Failure Center. There, they undergo standardized routine clinical re‐evaluation including electrocardiogram, transthoracic echocardiogram, and six‐minute‐walk test, as well as blood sampling for immediate analysis and long‐term storage. On these occasions, participants will also complete self‐reported questionnaires regarding health‐related quality of life, depression, cognitive abilities, and report HF‐related resource utilization. Further, hospitalizations experienced since the last visit are documented. Hospital discharge letters are requested from respective hospitals or sought for by general practitioners, cardiologists, or patient relatives. In case of fatalities occurring between follow‐up visits, the circumstances of death are clarified from death certificates, hospital letters, or reports from the attending physicians and relatives.

If patients do not attend outpatient follow‐up visits within one month of the scheduled date, the respective information is obtained using a standardized telephone follow‐up by trained AHF‐ImmunoCS staff.

## Immunophenotyping

### Heart‐reactive antibodies

HRAs are determined in the prospectively collected blood samples taken at baseline, and 6 weeks (F6w) and six months later (F6). The first method we use to detect HRAs is an immunofluorescence test (IFT) kit with monkey heart slides as substrate and an anti‐human IgG‐FITC as secondary antibody as well as standard positive and negative controls. Sera are rated as negative, weakly positive, or positive, and the latter categories are considered “positive”. All slides are assessed independently by three observers for fluorescence of myofibrils. Congruent results of at least two observers will enter the analyses.

**Figure 2:**
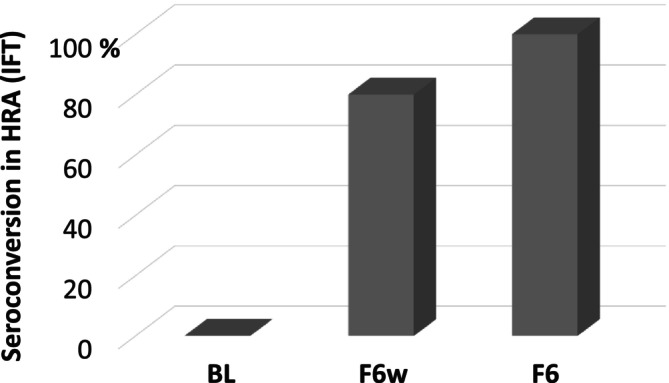
Timing of seroconversion in patients hospitalized for decompensated heart failure. In 10 patients negative for heart reactive antibodies (HRAs, measured by immunofluorescence test, IFT) at baseline (BL) but positive at 6 months follow‐up (F6), 80% showed seroconversion already after 6 weeks (F6w). An eleventh patient negative for HRA at BL who missed the F6w is not included in this figure.

In December 2023, a feasibility check was performed. Analysis of the first 59 patients (42% women, 72 ± 14 years) who had completed the 6‐month follow‐up period and hence had valid IFT test results from baseline, F6w, and F6 revealed the following:

- 21 patients were HRA‐positive at baseline;
- 38 patients were HRA‐negative at baseline;
- after six months, 11 patients (29%) had converted from HRA‐negative to HRA‐positive. In one patient, F6w status was missing. From the other 10 patients, 8 (80%) had seroconverted already after six weeks (*Figure* *2*).

Striated, that is, zebra crossing‐like, patterns on heart tissue slides may be caused by autoantibodies against, for example, titin, actinin, and/or FHL, and most likely reflect multiple reactivities. To detect HRAs not producing a striated pattern in the IFT and to gain knowledge on defined cardiac autoantigens recognized by HRAs, we set up a panel of six different bead‐based assays (*Table* *2*). Of the autoantigens studied by us, myosin light chain 7 and, in females, troponin I3 are exclusively expressed in the heart muscle according to the HUMAN PROTEIN ATLAS (*Table* *2*).

**Table 2:** Tissue selectivity of the cardiac autoantigens studied. The table summarizes protein expression according to the Human Protein atlas (https://www.proteinatlas.org)

Protein	Skeletal muscle	Smooth muscle	Heart muscle	Other tissues
MYH 7	**Yes**	No	**Yes**	No
MYH 6	**Yes**	No	**Yes**	No
MYL 7	No	No	**Yes**	No
Tropomyosin 1	**Yes**	**Yes**	**Yes**	**Yes**
Troponin I3	No	No	**Yes**	**Yes**
β1‐adrenergic receptor	No	No	**Yes**	**Yes**

Please see the Discussion section for a reflexion on the significance of the reported differences in antigen expression in these tissues.

MYH, myosin heavy chain; MYL, myosin light chain.

## Statistical considerations

### Endpoints

The primary endpoint is defined as the incidence of events (HF re‐hospitalization or all‐cause death) occurring during the observation period. This endpoint will be evaluated in the total cohort and separately in subgroups of particular interest (e.g., subgroups according to sex, to LVEF as well as to aetiology of heart failure).

Secondary endpoints (if applicable: cross‐sectionally and changes over time)

- Adaptive immune response pattern at baseline, week 6 (F6w) as well as at month 6 (F6), month 12 (F12), and month 18 (F18). We will profile: HRAs and autoantibodies against defined cardiac antigens including titers and quality, in‐depth immunophenotyping of peripheral blood leukocyte subsets
- Cytokine profile, inflammation markers (CRP, IL‐1β, IL‐2, IL‐4, IL‐5, IL‐6, IL‐8, IL‐10, IFNγ, TNFα, IL12p70, IL‐17A) in the acute in‐hospital phase and at F6w, F6, F12 and F18.
- Cardiac structure and function (echocardiography; left and right ventricular size and wall thickness, LVEF, right ventricular (RV) fractional area change, LV and RV systolic and diastolic deformation and deformation velocity, left atrial size and function) at F6w, F6, F12, and F18
- HF severity (NYHA functional class, N‐terminal pro‐B type natriuretic peptide level, 6‐minute walking distance) at F6w, F6, F12, and F18
- Hospitalization (for worsening HF and all‐cause, respectively) at F6, F12, and F18
- Death (cardiac and all‐cause, respectively) at F6, F12, and F18

### Sample size and power calculation

The initial sample size was calculated based on the results of the pilot study.^29^ We aimed to power the current study to quantify meaningful differences in the primary end point at F18 with respect to the development of HRAs after an episode of AHF‐related decompensation (software: Paths11). In our pilot study, 5 out of 15 (33.3%) patients with newly developed HRAs (group A), 1 out of 11 (8.3%) patients with persistently negative HRAs (group B), and 2 out of 21 (9.5%) patients with persistently positive HRAs (group C) reached the primary end point death or hospitalization for HF, respectively. Hence, regarding the primary endpoint, there was a difference between patients with newly developed HRAs and the other two patient groups of 25% and 23%, respectively. Targeting a power of 90% and an alpha of 5%, we needed at least 56 patients in the smallest patient group (here group B), resulting in a factor of 5.1 (56 = 11 * 5.1). On a conservative basis, we used the factor 5.5. Assuming a similar allocation into the respective groups, this had resulted in sample sizes *n* = 83 for group A, *n* = 61 for group B, and *n* = 116 for group C, that is, 260 patients in total. Based on prior experience, we assumed a dropout rate of 10% (*n* = 26 patients) up to F6w, that is, the earliest time point to assess changes in the adaptive immune response to AHF. We therefore aimed for a total sample size of 286 patients.

In December 2023, a feasibility check was performed. Patient recruitment followed the aspired recruitment targets, resulting in a patient sample of *n* = 259 patients (*Table* *3*). Analysis of the first 59 patients who had completed the 6 months follow‐up revealed the following distribution: group A 19%, group B 46%, and group C 35%. Further, there was no substantial change in the prevalence of HRAs between F6w and F6 (*Figure* *2*). Hence, in patients who had missed F6w, the F6 test result can be used with sufficient certainty to allocate a patient to one of the three groups.

**Table 3:** Characteristics of all patients included in the AHF‐ImmunoCS until 31 December 2023

Number of patients	259
Women	99 (38)
Age (years)	72 (13)
NYHA III/IV	221 (58)
De novo heart failure	97 (37)
LVEF (%)	50 (35; 56)
LVEF category	
≤40%	88 (34)
41–49%	40 (15)
≥ 50%	131 (51)
NT‐proBNP (pg/mL)	4,322 (1986; 10,591)
NT‐proBNP ≥ 125 pg/mL	258 (99)
Cause of heart failure	
Ischaemic heart disease	92 (36)
Cardiomyopathy	36 (14)
Hypertensive heart disease	28 (11)
Rhythm disorders	37 (14)
Valvular heart disease	37 (14)
Myocarditis	4 (2)
Storage disease	7 (3)
Other	18 (7)
Pharmacotherapy	
ACEi/ARB/ARNi	196 (76)
Beta‐blocker	227 (88)
MRA	133 (51)
SGLT2i	178 (69)
Glycoside	27 (10)
Antihypertensive drug	57 (22)
Thiazide	11 (4)
Loop diuretic	227 (88)
Steroid	15 (6)
Lipid lowering drug	160 (62)
Platelet aggregation inhibitor	92 (36)
Anticoagulant	164 (63)
Antidiabetic drug (including insulin)	81 (31)

Numbers are *n* (%) or median (quartiles), as appropriate.

ACEi, angiotensin conversion enzyme inhibitor; ARB, angiotensin receptor blocker; ARNi, angiotensin receptor blocker neprilysin inhibitor; LVEF; left ventricular ejection fraction; MRA, mineralocorticosteroid receptor antagonist; NT‐proBNP, N‐terminal pro B‐natriuretic peptide; NYHA, New York Heart Association functional class; SGLT2i, sodium glucose co‐transporter 2 inhibitor.

Further, we observed a higher than anticipated dropout rate: out of the first 100 patients recruited, 75 attended F6w, 68 attended F6, and 81 attended at least one of either visit. The respective reasons for non‐attendance are displayed in *Table* *4*. Hence, we had to calculate with a 20% dropout rate.

**Table 4:** Reasons for non‐attending the follow‐up visit 6 weeks after the index hospitalization in the first 100 patients included in the AHF‐ImmunoCS

	Number of patients
Prolonged or re‐hospitalization	4
Rehabilitation	3
Logistic reasons	3
Incompliance	2
Death	4
Withdrawal of consent	3
Perceived overburdening	2
Perceived high risk for infection with SARS‐CoV2	1
Perceived weakness	2
Care for sick relatives	1

Based on these observations, the sample size was re‐calculated as follows: In line with the original power calculation, we aimed for at least 61 patients in the smallest patient group. Assuming a prevalence of 20% for the smallest patient group (currently the smallest group is group A with 19%), the aspired total patient number with a valid HRA test at baseline and F6w or F6 is *n* = 305 (61 divided by 0.20). Assuming a dropout rate of 20%, the aspired total sample size has been estimated at 381 patients.

## Discussion

Based on sound methodology, the AHF‐ImmunoCS prospective cohort study will substantially contribute to the understanding of characteristics, determinants and the pathophysiologic role of the adaptive immune response following an AHF event. Embedded into a consortium of basic and clinical researchers united in the Collaborative Research Centre CRC 1525, different aspects of the adaptive immune response following an AHF hospitalization will be observed and analysed.

So far, our cohort reflects a typical real‐world collective of AHF patients showing an even distribution of HFrEF and HFpEF and a substantial frequency of *de novo* HF. Further, we were able to recruit a relatively high proportion of women, and our AHF patients exhibit a fairly typical variation in the causes of HF, allowing us to evaluate the role of the adaptive immune system in different disease entities. Comprehensive serial clinical phenotyping of our patients will enable us to determine potential effects of differential immunological phenomena.

Concordant with the results of our pilot study, we identified three distinct patient subgroups based on IFT results. In one subgroup, HRAs detected by IFT were present already at baseline. In our pilot study, the prognosis of such patients was equal to the prognosis of another subgroup comprising patients with persistently negative results for HRAs in the IFT, which would be in line with the existence of ‘harmless’ HRAs. Binding of defined cardiac antigens by HRAs in the bead assays established by us might clarify whether there are ‘harmless’ and ‘harmful’ HRAs in HF patients and whether they can be identified according to the cardiac antigens they recognize. This hypothesis deserves further investigation and comparison with population‐based data.

Further, in line with our pilot data, we found a subgroup of patients with newly developing HRAs after an episode of AHF‐related hospitalization. The majority of these patients exhibited HRAs already at six‐week follow‐up, supporting the hypothesis of a causal connection between AHF and an adaptive immune response.

Apart from the IFT, we are also using a set of bead‐based assays detecting HRAs against defined cardiac antigens (*Table* *2*). Myosin light chain 7 and, in females, troponin I3 are expressed only in the heart muscle. The situation is less clear for myosin heavy chain 6, which, apart from the heart muscle, may also be expressed in skeletal muscle. However, the concentration of myosin heavy chain 6 mRNA compared to protein expression is conspicuously low in skeletal muscle. In addition, myosin heavy chains 6 and 7 are 93% identical at the amino acid level according to Uniprot, meaning that cross‐reactivity of the reagents used for detecting myosin heavy chain 6 in skeletal muscle might account for the reported expression.

Detailed analysis of HRA reactivities in patients considered to be at highest risk of poor disease outcome, according to our pilot data, will reveal whether prognostic HRA patterns can be defined and used as guidance for therapeutic decisions. Apart from evaluating HRA patterns for prognostic purposes, the pathophysiological role of the different HRA entities will also be the subject of our future research.

In summary, AHF‐ImmunoCS was embedded into the strong research environment of CRC 1525, providing ideal conditions to follow up on the study's rationale and to apply state‐of‐the‐art methodology. A first evaluation in December 2023 showed consistent recruitment of a representative HF patient cohort. Preliminary immunologic analyses confirm our pilot data and extend previous findings by revealing that, in the vast majority of sera from high‐risk patients, HRAs could already be detected six weeks after the acute event. Hence, AHF‐ImmunoCS can be expected to substantially advance our understanding of the immune system in AHF progression by addressing the primary hypothesis that induction of HRAs after AHF hospitalization is associated with adverse prognosis. In addition, we hope that findings from this study will allow us to identify high‐risk patients early, generate hypotheses for future investigations, and outline novel immunotherapeutic concepts for AHF.

## Conflict of interest

C. Morbach reports research cooperation with the University of Würzburg and Tomtec Imaging Systems funded by a research grant from the Bavarian Ministry of Economic Affairs, Regional Development and Energy, Germany (MED‐1811‐0011 and LSM‐2104‐0002); she is supported by the German Research Foundation (DFG) within the Comprehensive Research Center 1,525 ‘Cardio‐immune interfaces’ (453,989,101, project C5) and receives financial support from the Interdisciplinary Center for Clinical Research ‐ IZKF Würzburg (advanced clinician–scientist program; AdvCSP 3). She further received advisory and speakers' honoraria as well as travel grants from Tomtec, Edwards, Alnylam, Pfizer, Boehringer Ingelheim, Eli Lilly, SOBI, AstraZeneca, NovoNordisk, Alexion, Janssen, Bayer, Intellia, and EBR Systems; she serves as principal investigator in trials sponsored by Alnylam, Bayer, NovoNordisk, Intellia, and AstraZeneca.

## Funding

This work was supported by the German Research Foundation (DFG) within the Comprehensive Research Center 1525 ‘Cardio‐immune interfaces’ (453989101, project C5). Further, CM receives financial support from the Interdisciplinary Center for Clinical Research ‐ IZKF Würzburg (advanced clinician–scientist program; AdvCSP 3).

## Appendices

none.
